# Investing preventive care and economic development in ageing societies: empirical evidences from OECD countries

**DOI:** 10.1186/s13561-021-00321-3

**Published:** 2021-06-04

**Authors:** Fuhmei Wang, Jung-Der Wang

**Affiliations:** 1 Department of Economics in College of Social Science and Department of Public Health in College of Medicine, National Cheng Kung University, Tainan, Taiwan; 2 Department of Public Health, National Cheng Kung University College of Medicine, Tainan, Taiwan; 3 Department of Occupational and Environmental Medicine, National Cheng Kung University Hospital, College of Medicine, National Cheng Kung University, Tainan, Taiwan

**Keywords:** Ageing, Prevention, Economic growth, OECD countries

## Abstract

**Background:**

The proportion of the elderly aged 65 years old or above will reach 16% in 2050 worldwide. Early investment in effective prevention would generally reduce the morbidity, complication, functional disability, and mortality of most chronic illnesses and save resources in both healthcare and social services. This research aims to investigate how the optimal allocation of medical resources between prevention and treatment adds value to the population’s health as well as examine the interaction between ageing, health, and economic performance.

**Methods:**

This research undertakes ageing-health analyses by developing an economic growth model. Based on the Organization for Economic Co-Operation and Development (OECD) countries’ experiences over the period from 2000 to 2017, this research further examines the hypothesis that an ageing society could increase demand for preventive and curative healthcare.

**Results:**

Theoretical analysis found that the prevention share for maximizing growth is the same as that for minimizing ill health and maximizing welfare; this share increases with treatment share and ageing ratios. Estimation results from OECD countries’ experiences indicate that when treatment share increases by 1%, the prevention demand increases by 0.036%. A one-percent increase in the ageing ratio yields a change in prevention share of 0.0368%. The optimal share of prevention health expenditure to GDP would be 1.175% when the prevalence rate of ill health isat 6.13%; a higher or lower share of prevention would be accompanied with a higher prevalence of ill health. For example, a zero and 1.358% preventive health expenditure would be associated with an 18.01% prevalence of ill health, while the current share of prevention of 0.237% is associated with a 10.26% prevalence of ill health.

**Conclusion:**

This study shows that appropriate prevention is associated with decreases in the prevalence rates of ill health, which in turn attains sustainable growth in productivity. Too much prevention, however, could lead to higher detection of new chronic diseases with mild severity, which would result in longer illness duration, and higher prevalence rates of ill health. With suitable allocation of medical resources, the economic growth rate will help to cancel out increases in healthcare spending for the elderly and for expenses needed for the improvement of the population’s health as a whole.

## Background

The proportion of the elderly aged 65 years old or above will reach 16% in 2050 worldwide [1]. As people age, they develop multiple comorbidities of chronic diseases. Effective prevention would reduce the number of new cases, complications, and functional disabilities, which would slow down the demand of healthcare resources. Many studies have worked on the theoretical model of longevity [2, 3] and health risk [4, 5]. Murphy and Topel utilized a framework to estimate improvements in health and calculate the social benefits of longevity (ageing) in the United States [6]. Hall and Jones investigated the utility effects of longer life expectancy, which is closely related to aging issues [7]. These studies contain information on the macroeconomic effects of ageing in the population¸ although preventive interventions were not mentioned. This study will demonstrate the macroeconomic effects of prevention on an ageing society.

Medical care spending devoted to the elderly are expected to rise continually. Researches have highlighted the impact of ageing on healthcare spending and urged governments to focus on prevention and treatment [8, 9]. Healthcare accounts for a huge part of government fiscal expenditure. For example, the average health and long-term care expenditure is expected to reach about 13% of the GDP by 2050 in Organization for Economic Co-Operation and Development (OECD) countries [10]. Whether increased health expenditure has positive aggregate impacts holds important implications to policymakers.

Because of the pressure which a rapidly ageing population places on limited medical resources, policymakers and psychiatric consultants are beginning to pay more attention to the issue of prevention. The effectiveness of prevention on the occurrence of disease-related events among targeted populations have been investigated [10–14]. However, few studies have modelled and quantified the potential economic consequences of preventive care in ageing societies. Several studies have reported the link between prevention and health and economic performance but ageing country experiences were not taken into consideration [15, 17]. Countries might include different types of expenditures on prevention; those listed by the OECD are generally comparable and policy relevant. Following the classification of OECD health statistics, this research defines prevention/public health and administration expenditure as prevention expenditure [15]. Total health expenditure minus preventive expenditure would be the expenditures for diagnosis, treatment, and rehabilitation, which will be termed as “treatment expenditure” in this article. Based on OECD countries’ experiences, the present work investigates how the optimal allocation of medical resources adds value to the population’s health as well as examines the interaction between ageing and health, presented by the prevalence rates of ill health and economic performance.

This research contributes to and improves upon earlier studies in the following ways: First, this study is among the first to distinguish between prevention and treatment spending and integrate concerns of ageing and prevalence rates of ill health to investigate the effectiveness of medical resource allocation in a growing economy. Second, this research endeavors to find the optimal amount of preventive healthcare to be provided to improve the population’s health and productivity. Third, we quantified the analytical effects of health interventions through empirical estimations from OECD countries’ prevention experiences.

## Methods

This research uses a discrete-time version of growth model with infinitely lived aged population. The theoretical model could be regarded as a simplified structure of general equilibrium macroeconomic model and only incorporates goods market with government and households. General equilibrium models in the microeconomic tradition typically involve a multitude of different goods markets. The theoretical model incorporates the concept of longevity [2, 3, 7] and health risk [4, 5] to build in a manner that practically can be used to depict any type of economy. Based on OECD country experiences, we examine whether the results receive further empirical support through estimation procedures, as a robustness check. This not only will re-validate the original findings, but also will provide sound empirical support to the validity of the theoretical model.

### The theoretical model

Consider an economy that is populated by households and firms. The representative household possesses the following discounted sum of future instantaneous utilities from consumption *C*_*t*_, and good health, (1-*R*_*t*_)*H*_*t*_:

1 $$ {\sum}_{t=0}^{\infty}\frac{1}{{\left[1+\left(1/\chi \right)\right]}^t}\left[1\mathrm{n}\ {C}_{\mathrm{t}}\;\eta\;1\mathrm{n}\left(1-{R}_t\right){H}_t\right] $$

The health condition is uncertain with the prevalence rate of ill health, *R*_*t*_. The representative household at a specific age has a life expectancy of χ; the reciprocal of this parameter 1/χ, presents an indication of the mortality rate and the subjective discounting rate [7]. Parameter η presents the positive impact of health on utility. The instantaneous output per capita, *y*_*t*_, is produced using the instantaneous private capital input per capita, *k*_*t*_, and the instantaneous public non-health expenditure, *g*_*t*_, according to the following production technology:

2 $$ {y}_t={Ak}_t^{\alpha }{g}_t^{1-\alpha } $$

in which *A* is the technological parameter. Parameters *α* and (1-*α*) are the shares and contributions of private capital and public non-health capital in goods production. All producers set the same price and produce output in equilibrium in a perfectly competitive goods market. Part of the productions are devoted to healthcare expenditure for improving the population’s health, which in turn leads to increases in household utilities [7]. The health expenditure spent on diagnosis and treatment is proportional to the output with the proportion of *T*. Individuals demand curative healthcare services to maintain health. Longer life expectancy is associated with better health status [18].

3 $$ {H}_t=\chi {Ty}_t $$

Most of the OECD countries adopt universal health coverage (UHC) systems. The amount the government spends for preventive healthcare is a fixed fraction of the output, *P*, in order to reduce the probability of ill health or the prevalence rates of ill health. Early preventive health intervention may possibly decrease the occurrence of catastrophic illnesses in the long run for the population [11]. The future prevalence rate of ill health would be inversely correlated to current preventive health expenditure.

4 $$ {R}_{t+1}=1/{Py}_t $$

The household’s budget constraint is:

5 $$ {k}_{t+1}={k}_t+\left(1-\tau -\theta \right){y}_t-{C}_t $$

Variable *k*_t_ is the physical capital at the beginning of the period, *τ* is the income tax rate, and *θ* is the health insurance payments in the universal health coverage (UHC) system. Initial capital plus savings, the disposable income that is not consumed, presents the evolution of capital stock at period *t* + 1, *k*_t + 1_. The public-sector balanced budget constraint is:

6 $$ {g}_t+{Ty}_t+{Py}_t+\frac{T}{P}{\varepsilon}^2{Ty}_t=\left(\tau +\theta \right){y}_t $$

The government collects income taxes, *τy*_*t*_*,* and health insurance premiums*, θy*_*t*_*,* to finance productive expenditures, *g*_*t*_, allocate medical expenditures to curative and preventive services, *Ty*_*t*_ and *Py*_*t*_, in order to satisfy the population’s healthcare demands and devote medical spending on the elderly (*ε*^2^*Ty*_*t*_)*T*/*P*. Chronic illnesses are prevalent in old age and healthcare treatment expenditures inevitably increase with the ageing ratio, *ε*^2^*Ty*_*t*_. With adequate provision of preventive healthcare, expenditures on illnesses diagnosed at older ages could be reduced or avoided. Healthcare spending in response to an ageing population decreases with preventive health services. Regarding the age profile, this spending increases as the demand for curative services grows, as the fourth term on the left-hand side of Eq. 6 shows. Rearranging government budget constraints, Eq. 6, along with the production function, Eq. 2, yields.

7 $$ \frac{g}{k}={A}^{\frac{1}{\alpha }}{\left(\tau +\theta -T-P-\frac{T^2{\varepsilon}^2}{P}\right)}^{\frac{1}{\alpha }} $$

The representative household chooses consumption and owns good health stock to maximize the discounted sum of utilities defined in Eq. 1 subject to Eq. 5. The first-order conditions are given by Eqs. 8, 9, 10 and 11.

8 $$ \frac{1}{C_t{\left[1+\left(1/\chi \right)\right]}^t}={\lambda}_t $$

9 $$ \frac{\eta }{\left(1-{R}_{t+1}\right){\left[1+\left(1/\chi \right)\right]}^{t+1}}=\frac{\lambda_t}{R_{t+1}^2} $$

10 $$ \frac{\eta }{H_t{\left[1+\left(1/\chi \right)\right]}^t}=\frac{\left(1+\theta \right){\lambda}_t}{\chi } $$

11 $$ \frac{\lambda_t}{\lambda_{t+1}}=1+\left(1-\tau -\theta \right)\alpha A{\left(\frac{g}{k}\right)}^{1-\alpha } $$

12 $$ \frac{R_{t+1}}{R_t}={\left\{\frac{1}{\left[1+\left(1/\chi \right)\right]}\left[1+\left(1-\tau -\theta \right)\alpha {A}^{\frac{1}{\alpha }}{\left(\tau +\theta -T-P-\frac{T^2{\varepsilon}^2}{P}\right)}^{\frac{1-\alpha }{\alpha }}\right]\right\}}^{-1/2} $$

13 $$ \underset{t\to \infty }{\lim}\frac{1}{{\left[1+\left(1/\chi \right)\right]}^t}{\lambda}_t{k}_t=0 $$

In these equations, *λ*_*t*_ is the Lagrange multiplier and presents the shadow value of the private capital stock assessed in utility terms at time *t*. Using Eqs. 9 and 11, Eq. 12 characterizes the evolution of the prevalence rates of ill health. Eq. (13) is the transversality condition and states that it cannot be optimal to hold capital forever. Combining Eq. 7 with Eqs. 8 and 11, generates the growth rate of consumption:

14 $$ \gamma =\frac{1}{\left[1+\left(1/\chi \right)\right]}\left[1+\left(1-\tau -\theta \right)\alpha {A}^{\frac{1}{\alpha }}{\left(\tau +\theta -T-P-\frac{T^2{\varepsilon}^2}{P}\right)}^{\frac{1-\alpha }{\alpha }}\right]-1 $$

In the standard one-sector endogenous growth model, consumption *C*, physical capital, *k*, and output, *y,* all grow at a constant rate. The economic growth rate depends on the net marginal productivity of capital per capita, as well as life expectancy, and the ageing ratio. The framework has considered the social planner objective and the goods market is certainly satisfied through incorporating the household budget constraint in Eq. 5 and the government budget constraint in Eq. 6, together with the production function in Eq. 2. Furthermore, we are interested in whether the growth-maximization equilibrium in an ageing economy leads to a desirable outcome. Taking into account the best healthcare resource allocation and maximizing Eq. 14 with respect to the prevention share, *P* generates Eq. 15:

15 $$ \frac{\partial \gamma }{\partial P}=\frac{\left(1-\alpha \right)\left(1-\tau -\theta \right)\alpha {A}^{1/\alpha }}{\alpha \left[1+\left(1/\chi \right)\right]}{\left(\tau +\theta -T-P-\frac{T^2{\varepsilon}^2}{P}\right)}^{\frac{1-2\alpha }{\alpha }}\left[\left({T}^2{\varepsilon}^2/{P}^2\right)-1\right)\Big]{\displaystyle \begin{array}{c}>\\ {}=0\kern0.5em \mathrm{if}\\ {}<\end{array}}{\displaystyle \begin{array}{c}<\\ {}P=\kern0.5em \\ {}>\end{array}} T\varepsilon $$

The influence of prevention on economic performance is ambiguous since the provision of preventive healthcare maintains or improves the population’s health status but crowds out the medical resources allocated to curative healthcare and harms health stock. The former leads to positive productivity effects and the latter is associated with negative economic performance. A nonlinear nexus between prevention expenditure share and economic growth is presented. A bigger prevention share is associated with better economic performance. However, after a critical prevention share, negative impacts on economic performance are expected, which has been noticed in a previous study [15]. In an ageing economy, Eq. 15 implies that in order to attain economic growth, the optimal share of preventive health expenditure relative to output increases with diagnosis and treatment share as well as with ageing ratios.

16 $$ {P}^{\ast }= T\varepsilon $$

Older people generally have greater healthcare needs than younger people, leading to increases in the amount of prevention expenditure demanded. Preventive interventions do not intend to sacrifice diagnosis and treatment expenses that are valuable in restoring health stock. In fact, patients with ill health status could be converted back to either complete recovery from acute infection(s) or injury or stabilized condition for chronic diseases, such as hypertension, diabetes, etc. Simultaneous increases in both expenditures of prevention and treatment are designed to improve health with higher productivity and faster economic growth. Prevention could be regarded as a complement but not a substitute for curative health services.

We further analyzed the influences of medical resource reallocation between preventive and curative healthcare expenditure on social welfare, which is specified as the summation of the utilities of all the individuals’ utility functions in the society [16]. Given the initial private capital stock *k*_*0*_, both private consumption and health stock grow at a constant rate γ along the balanced growth path:

17 $$ {C}_t={C}_0{\left(1+\gamma \right)}^t $$

18 $$ {H}_t={H}_0{\left(1+\gamma \right)}^t $$

For a given value of P^*^, Eq. 16, and the evolution of consumption, Eq. 17, this research calculated the initial values of consumption and health stock from Eqs. 2, 5, 7, and 14:

19 $$ {C}_0={k}_0\left[\left(1-\frac{\alpha \chi}{1+\chi}\right)\left(1-\tau -\theta \right){A}^{\frac{1}{\alpha }}{\left(\tau +\theta -T-P-\frac{T^2{\varepsilon}^2}{P}\right)}^{\frac{1-\alpha }{\alpha }}+\frac{1}{1+\chi}\right] $$

20 $$ {H}_0=\frac{\chi \eta {k}_0}{1+\theta}\left[\left(1-\frac{\alpha \chi}{1+\chi}\right)\left(1-\tau -\theta \right){A}^{\frac{1}{\alpha }}{\left(\tau +\theta -T-P-\frac{T^2{\varepsilon}^2}{P}\right)}^{\frac{1-\alpha }{\alpha }}+\frac{1}{1+\chi}\right] $$

Using Eqs. 8, 9, 11, and 19 yields the equilibrium prevalence rate of ill health or disease:

21 $$ {R}_0=\frac{-\left[1+\left(1/\chi \right)\right]+\sqrt{{\left[1+\left(1/\chi \right)\right]}^2+4\eta {C}_0\left[1+\left(1/\chi \right)\right]}}{2\eta {C}_0}{\left(1+\gamma \right)}^{\frac{1}{2}} $$

In order to minimize the prevalence rate of disease, we differentiated Eq. 21 with respect to the prevention ratio to yield:

22 $$ \frac{\partial {R}_0}{\partial P}=\frac{1}{4}{R}_0\frac{1-\alpha }{1+\gamma}\left(\frac{1+\chi }{\chi}\right){\left(\frac{1+\gamma \chi +\gamma }{\chi}\right)}^{\frac{1-2\alpha }{1-\alpha }}{\left(1-\tau -\theta \right)}^{\frac{3\alpha -1}{\alpha }}{\alpha}^{\frac{2\alpha -1}{\alpha }}{A}^{\frac{3\alpha -1}{\alpha^2}}\left({T}^2{P}^2{\varepsilon}^2-1\right){\displaystyle \begin{array}{c}>\\ {}=0\kern0.5em \mathrm{if}\\ {}<\end{array}}{\displaystyle \begin{array}{c}<\\ {}P=\kern0.5em \\ {}>\end{array}} T\varepsilon $$

Equation 15 along with Eq. 22 imply that when an economy introduces growth-maximizing prevention healthcare with the share P^*^, the positive impacts rise through reducing the prevalence rates of ill health and hence raises productive capacity. The positive relationship between health services, the health of a nation, and its economic prosperity is well recognized [19]. Substituting Eqs. 19 and 20 into Eq. 1, yields the households’ welfare function of *P* over an infinite planning horizon:

23 $$ U=\left(1+\chi \right)\left(\ln {C}_0+\eta \ln {H}_0\right)+\chi \left(1+\chi \right)\left(1+\eta \right)\ln \left(1+\gamma \right)-\left[\eta {R}_0\left(\frac{1+\chi }{1-\chi \gamma}\right)+\frac{\eta {R}_0^2}{2}\left(\frac{1+\chi }{1-2\chi \gamma -{\chi \gamma}^2}\right)\right] $$

Equations 22 and 23 indicate that appropriate allocation of medical resources between treatment and prevention could lead to welfare improvements through decreasing the prevalence rates of ill health.

### Empirical application: estimating the quantitative influences of ageing on the prevention healthcare demanded

#### The link between ageing with prevention and treatment

This research has been built with a model that could practically be applied to any type of economy. Therefore, we would like to examine whether the theoretical results could receive further empirical support. We examined the model’s predictions for ageing and treatment expenditure on preventive healthcare provision. Based on OECD countries’ experiences, the role of preventive and curative health spending in economic performance has been demonstrated in a previous study [17]. OECD official health statistics of public health have been published since 1995. In order to compile complete and longitudinal data, datasets of ageing, prevention, and treatment variables in 36 OECD member countries were observed from 2000 to 2017 and collected from OECD statistics. The mean values of the focus variables were: prevention share of GDP rose from 0.204% in 2000 to 0.237% in 2017; treatment share of GDP rose from 7.33% in 2000 to 8.26% in 2017; ageing ratio rose from 12.77% in 2000 to 16.62% in 2017; prevalence rates of ill health rose from 5.13% in 2000 to 10.26% in 2017. Analytical results through model construction show a strong link between prevention and treatment and ageing ratio. The corresponding regression result is:

24 $$ {\displaystyle \begin{array}{l} Prevention\ share=-0.056+0.036\ \mathrm{treatment}\ \mathrm{share}\\ {}\left(t/ SE=-3.07/0.018\right)\ \left(t/ SE=16.53/0.002\right)\\ {}\mathrm{N}=543\kern0.5em {\mathrm{R}}^2=0.34\end{array}} $$

The abbreviations *t* and *SE* in the parentheses of Eq. 24 respectively present *t* statistics and standard error. When treatment share increases by 1%, the prevention share increases by 0.036%. About 34% of the variance in prevention allocation in OECD countries can be explained by variations in curative spending when making international comparisons. The intercept and the coefficient of treatment share are significant at a level of 0.1%. Furthermore, the estimated result for the interaction of ageing and curative spending on prevention is:

25 $$ {\displaystyle \begin{array}{l} Prevention\ share=0.133+0.0008\ \left(\mathrm{ageing}\ \mathrm{share}\times \mathrm{treatment}\ \mathrm{share}\right)\\ {}\left(t/ SE=10.88/0.012\right)\ \left(t/ SE=9.46/0.00\right)\\ {}\mathrm{N}=543\kern1em {\mathrm{R}}^2=0.14\end{array}} $$

In the presence of the interaction term of treatment by ageing, a one-percent increase in the ageing ratio yields a change in prevention share of (0.0008 + 0.036) = 0.0368%. The intercept and the coefficient of (*ageing share×treatment share*) are significant at a level of 0.1%. A strong statistical association exists between the interaction of ageing and curative spending and with prevention. Eqs. 24 and 25 show positive effects of ageing on the demand for curative and preventive healthcare.

## Results

### Optimal prevention for minimizing the prevalence rates of ill health

Based on Eqs. 16 and 22, we understand that optimal prevention share does exist for maximizing growth and welfare as well as minimizing the prevalence rates of ill health. The relationship between the prevalence rates of ill health and prevention is estimated as:

26 $$ {\displaystyle \begin{array}{l} Prevalence\ rates\ of\  ill\  health=18.007-38.369\ \mathrm{prevention}\ \mathrm{share}+24.049\ {\mathrm{prevention}\ \mathrm{share}}^2\\ {}\left(t/ SE=20.77/0.867\right)\ \left(t/ SE=2.59/9.269\right)\ \left(t/ SE=-6.27/6.115\right)\\ {}N=404\ {\mathrm{R}}^2=0.33\end{array}} $$

**Fig. 1:**
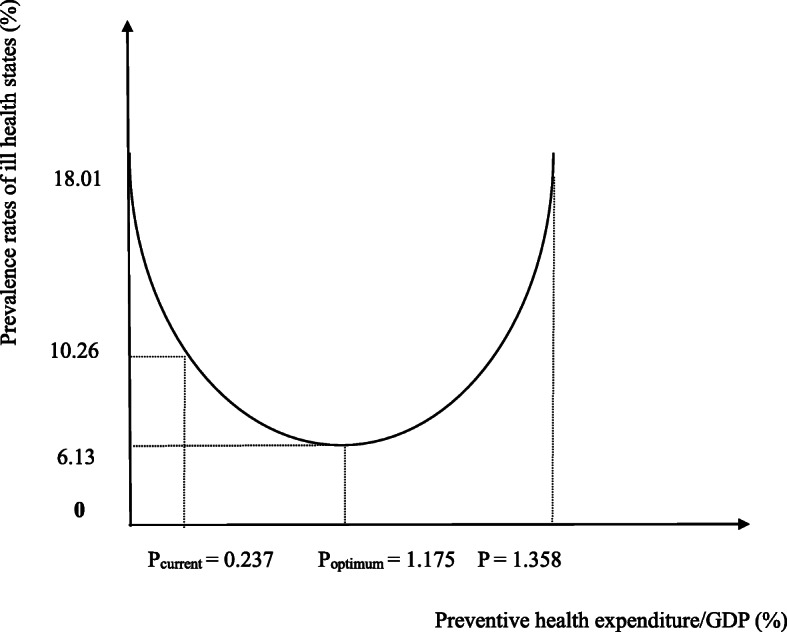
The relationship between prevention health expenditure and prevalence rates of ill health

Using Eq. 26, the current prevention share is 0.237% and the prevalence rate of ill health is 10.26%. Based on Eq.16, the optimal share of prevention health expenditure to GDP is 1.175%. Therefore, current prevention provision is under-provided. Figure 1 presents that under the optimum, the prevalence rate of ill health would be 6.13%; with zero preventive health expenditure or at a share of 1.596%, the prevalence rate of ill health would be 18.01%; when the real share is 0.237%, the incidence rate of disease would be 10.26%. Increasing spendings on prevention reduces the prevalence rates of ill health states, while improving the population’s health, raising the economy’s productivity, and enhancing the population’s welfare as the theoretical analysis and estimations show. However, after the optimum, raising preventive spending could crowd out curative expenditure and possibly increase the prevalence rates of ill health.

### The U-shape relationship between preventive health expenditure and prevalence rates of ill health

The prevalence rates of ill health are influenced by both the incidence rates of new cases and the mean duration of the diseases. One of the effective strategies for preventing most chronic diseases such as cancer, diabetes, hypertension, chronic obstructive pulmonary disease, cirrhosis of liver, etc. is to early diagnose and treat the disease. Although spending lots of resources on prevention may detect ill health at early stages when diseases are more easily controlled, this may cause more people to be diagnosed and survive with the diseases, resulting in higher prevalence rates. For example, understanding of the pathophysiology of hypertension makes experts redefine the disease as blood pressure exceeding 130/80 mmHg for early control [20]. But the U.S. Department of Health and Human Services estimates that the new definition would probably increase about 30% of the prevalence of hypertension [21]. Simultaneously, since more expenditures would be spent to control blood pressure, such efforts would prevent complications and premature mortality and possibly save money. Similarly, screening for lung cancer with high resolution computed tomography would detect more cases at an early stage, improving survival, and increasing the prevalence [22]. On the other hand, excess early diagnoses might result in an elevation of false positive cases and more money will be spent on confirming the diagnoses. For example, mammography might detect false positive breast cancer that would require more expenditure on diagnosis, while more frequent imaging tests may also expose the screened population to higher radiation doses and lead to higher rates of over-diagnosis [23].

Based on Eq. 7, a $1 investment on prevention of pandemic acute infectious diseases, e.g. the coronavirus disease 2019 (COVID-19), might gain a $7 saving on future treatment expenditure. While the saving from acute infectious disease prevention may easily yield a return within a short period of time, say, 6–12 months, the possible saving from prevention of chronic diseases at a cost of $1 would save around $7 from a lifetime horizon if social impacts such as savings of productivity loss were also considered [24]. The public-sector budget is balanced in each period. The saved resources could be devoted to the establishment of public infrastructure, which in turn leads to increased production and better economic performance as Eqs. 2 and 7 show. Early investment in effective prevention would generally reduce the morbidity, complication, functional disability, and mortality of most chronic illnesses and save resources in healthcare, productivity, and social services [25].

### More preventive health services are demanded in ageing economies

The nonlinear prevention-growth nexus of OECD countries has been verified in a previous study and the optimal prevention share was 0.44% without considering the factor of ageing [17]. This study incorporates the concerns of ageing with prevalence rates of ill health to investigate the prevention optimum. The current research found that the optimal preventive share for maximizing growth and minimizing prevalence rates of ill health is much higher than that without considering ageing and the prevalence rates of ill health in OECD countries, which may not have been noticed in earlier works. The higher the ageing ratio, the more preventive services are needed. This could be a valuable and important impact since it will increase healthy longevity and may enable people to work for longer in order to fund their retirement, which in turn partially offsets the reduction in productivity within an ageing population. Another positive development is that younger adults with optimal preventive health services could maintain health. Health status is positively correlated with optimal preventive investment, which in turn improves the population’s productivity and economic growth. Theoretical and empirical results demonstrate current policies at the macroeconomic level allows policymakers to properly allocate scarce resources across preventive and curative care sectors in an ageing society.

## Discussions and policy implications

This research examines the hypothesis that an ageing society could place increasing demand on preventive and curative healthcare. Although we found a consistent positive correlation between ageing ratios and prevention provision, as well as the existence of an optimal prevention share for maximizing growth and minimizing prevalence rates of diseases from theoretical analysis, it does not necessarily imply that the aging society is linked to prevention, health, and economic performance. We have, however, the following arguments to corroborate the above hypothesis: First, this research uses data from OECD countries to corroborate the validity of our model. OECD statistics are comprehensive and the people in these countries typically have longer life expectancy, therefore the data would be representative of ageing and/or aged societies. The results on preventive and curative care did present the problem of increasing health spending in a continually ageing economy. Moreover, although there were variations on the prevalence rates of ill health among different countries ranging from 1.7 to 23.2%, repeated measurements across 18 years of observation plus modelling under mixed effects would largely control potential confounding by different understandings of ill health. We are confident that our estimation results can reflect the influences of prevention on health status, the prevalence rates of ill health as well as the demand for healthcare without bias. Second, prevention efforts aim to reduce the incidence of ill health for the overall population. We understand prevention could compete with the medical resources of individuals who require treatment and thereby harm the population’s health. We thus examined how the provision of prevention is linked to treatment with regard to the factor of ageing and what the optimal prevention share for maximizing growth and minimizing the prevalence rates of ill health is as Fig. 1 shows. Third, we estimated the effects of rising ageing ratios on preventive and curative care, which still corroborate our hypothesis. Therefore, we tentatively conclude that the demand for prevention in an ageing economy increases with ageing ratios and optimally providing preventive healthcare could improve health, through reducing prevalence rates of ill health, which in turn increases the population’s productivity, and it deserves further attention.

Our findings suggest that the difficulties associated with prolonged life and long term care expenditure in developing and developed countries might be mitigated through preventive healthcare in complement with treatment healthcare, as verified above. Our research findings are in line with those of Wang et al. [15] on the discussion of the existence of optimal prevention, although the previous study did not take into account the factor of ageing, the prevalence of ill health, and empirical evidences from various countries. Theoretical and empirical based policies, which allocate medical resources between different health services in ageing societies, could be used to reduce the prevalence of ill health, as is assured in most health-policy issues and has important implications for policymakers.

## Conclusions

This research proposes the hypothesis that an ageing society could place increasing demand on preventive and curative health care. The theoretical model endeavors to reveal the relevant real-world factors at a macroeconomic level to examine this hypothesis. However, a model still has restrictions and might not take all realities into account. The limitation in our study is that we regarded the households as relatively homogenous and the population faced the same risk of ill-health. With data collected from before 2019 when ill health mainly resulted from chronic diseases, these assumptions could be in line with reality. Namely, experiences of chronic disease prevention in OECD countries provide support for the theoretical analysis. However, when the new pandemic infectious disease of COVID-19 occurred after 2020, a more complex model would be needed to accommodate for transmission dynamics and mortality in the elderly.

Developing and developed economies have witnessed unprecedented growth in the ageing/aged population. However, as the number of elderly people has rapidly expanded, more medical expenditures have been spent, affecting economic growth. Thus, enhancement in productivity by keeping the workforce as healthy as possible is of vital importance. In our analysis, we applied the growth model and treated a society’s adequate investment in prevention as a strategy for maintaining the economy’s productivity in an ageing economy. With limited medical resources, preventive and curative health care may be substitutes for each other financially but are complements in terms of health promotion and maintenance. This study shows that appropriate prevention is associated with decreases in the prevalence rates of ill health, which in turn attains sustainable growth in productivity. Excess prevention, however, could lead to higher detection of new chronic diseases with mild severity, which would result in longer disease duration and higher prevalence rates of ill health. The U-shape relationship between prevention provision and the population’s health status, presented by the prevalence rates of ill health, does exist. With suitable allocation of medical resources, the economic growth rate will help to cancel out increases in healthcare spending for the elderly and for expenses needed for the improvement of the population’s health as a whole. Analytical results from the study also offer alternative scenarios for proactive measures that can be used to assess the effectiveness of other kinds of health intervention. This research assumes that prevention expenditure is funded by the government. In real life, individual people also partially contribute to their own expenditure on health prevention. Fortunately, most OECD countries adopt a universal health coverage system and the government budgets are reasonable. Future research could begin with extensions on agencies that could be allowed to invest in health capital in order to take control of their own prevention health expenditure. Health capital could also depreciate over time and different illnesses have different prevalence rates and hence prevention strategies vary. Models must adopt specific protective measures to avoid transmission such as wearing masks, social distancing and washing hands if pandemic infectious diseases occur.

## Data Availability

The data underlying the results presented in the study are from third party web resources and are available from the URL: https://stats.oecd.org/. The macroeconomic statistics are open for the public.

## Abbreviations

OECD: Organization for Economic Co-Operation and Development
UHC: universal health coverage
